# Effectiveness of human papillomavirus vaccine against cervical precancer in Japan: Multivariate analyses adjusted for sexual activity

**DOI:** 10.1111/cas.15471

**Published:** 2022-07-11

**Authors:** Risa Kudo, Masayuki Sekine, Manako Yamaguchi, Megumi Hara, Sharon J. B. Hanley, Megumi Kurosawa, Sosuke Adachi, Yutaka Ueda, Etsuko Miyagi, Sayaka Ikeda, Asami Yagi, Takayuki Enomoto

**Affiliations:** ^1^ Department of Obstetrics and Gynecology Niigata University Graduate School of Medical and Dental Sciences Niigata Japan; ^2^ Department of Preventive Medicine, Faculty of Medicine, Saga University Saga Japan; ^3^ Hokkaido University Center for Environmental and Health Sciences Sapporo Japan; ^4^ Departments of Obstetrics and Gynecology Osaka University Graduate School of Medicine Osaka Japan; ^5^ Department of Obstetrics and Gynecology Yokohama City University School of Medicine Yokohama Japan; ^6^ Center for Cancer Control and Information Services National Cancer Center Tokyo Japan

**Keywords:** cervical cancer, cytological abnormality, HPV infection, HPV vaccine, sexual activity

## Abstract

Japanese girls aged 12–16 years are offered free human papillomavirus (HPV) vaccination and cervical cancer screening is conducted with cytology and not HPV testing from the age of 20 years. So far, no study has analyzed the effect of HPV vaccination against cervical precancers considering HPV infection status and sexual activity. We aimed to analyze the vaccine effectiveness (VE) against HPV infection and cytological abnormalities, adjusted for sexual activity. This study comprised women aged 20–26 years who underwent cervical screening in Niigata. We obtained HPV vaccination status from municipal records and a questionnaire along with information concerning sexual activity. Of 5194 women registered for this study, final analyses included 3167 women in the vaccinated group (2821 vaccinated women prior to sexual debut) and 1386 women in the unvaccinated group. HPV 16/18 (0.2% vs 3.5%), 31/45/52 (3.4% vs 6.6%), and 31/33/45/52/58 (5.0% vs 9.3%) positive rates were significantly lower in the vaccinated group (*P <* 0.001). No women vaccinated before sexual debut had HPV 16/18‐related cytological abnormalities. VE for HPV 16/18 infection and high‐grade cytological abnormalities in women vaccinated prior to sexual debut were 95.8% (95% CI 81.9–99.0%; *P <* 0.001) and 78.3% (95% CI 11.3–94.7%; *P =* 0.033), respectively, in multivariate analyses adjusted for age and number of sexual partners. However, analyses of all vaccinated women did not show significant effectiveness against cytological abnormalities. Our results showed the effectiveness of HPV vaccine against high‐grade cervical cytological abnormalities and the importance of the vaccination before sexual debut.

## Graphical abstract

We found that among Japanese women aged 20‐26 years, a high vaccine effectiveness (VE) of >90% was observed concerning HPV 16/18 infection and that VE was nearly 80% for HSIL+ cytological abnormalities in the group vaccinated prior to sexual debut using real‐world data.

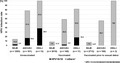

**Abbreviations**
aOR: adjusted odds ratio
ASC‐H: atypical squamous cells, cannot rule out high grade squamous intra‐epithelial lesion
ASC‐US: atypical squamous cells of undetermined significance
ASC‐US+: ASC‐US or worse
aVE: adjusted vaccine effectiveness
CI: confidence interval
HPV: human papillomavirus
HSIL: high‐grade squamous intraepithelial lesion
HSIL+: HSIL or worse
LSIL: low‐grade squamous intraepithelial lesion
NILM: Negative for intraepithelial lesion or malignancy
OR: odds ratio
SD: standard deviation
VE: vaccine effectiveness

## 1 INTRODUCTION

In Japan, cervical cancer affects approximately 11,000 women per year and approximately 2900 women die of it annually,^1^ with the number of patients and deaths gradually increasing. A rapid increase in the incidence of cervical cancer among young women has emerged as a social issue, particularly due to delayed marriage and childbirth.^2^, ^3^ The rate of cervical cancer screening, as a means of secondary prevention, remains low in Japan, at approximately 40%, as opposed to rates of 70%–80% in Europe and the United States.^4^, ^5^, ^6^, ^7^ The main method of primary prevention, namely, human papillomavirus (HPV) vaccination, began being publicly funded for 12–16‐year‐old girls in 2010 and was included in the national immunization program in April 2013. However, following several media reports of women experiencing various symptoms after vaccination, the Japanese Ministry of Health, Labour, and Welfare stopped actively recommending vaccination in June 2013, and the vaccination rate declined precipitously to 1%.^8^, ^9^, ^10^ The HPV vaccine is still included as a routine childhood vaccine and, while it is provided free of charge to girls of a determined age, coverage continues to be extremely low.^11^

Since the US FDA approved the HPV vaccine in 2006, national surveys globally have reported a decline in HPV infection rates,^12^, ^13^, ^14^ a decrease in the rate of cytological abnormalities,^15^ and a decline in the rate of histological abnormalities^16^, ^17^, ^18^ as a result of the HPV vaccine. A reduction in the prevalence of invasive cervical cancer was first reported in Sweden in 2020, which has been followed by other reports from Demark and England.^19^, ^20^, ^21^ In Japan, although a decrease in the rate of cytological and histological abnormalities due to HPV vaccination has been reported,^22^, ^23^, ^24^, ^25^, ^26^ many challenges remain in terms of conducting population‐based epidemiological research on the effectiveness of the HPV vaccine in Japan. First, >1700 individual municipalities individually manage vaccination records, and there is no national database. Second, reporting bias is an issue when relying on patient memory recall concerning vaccination history.^27^ Third, cervical screening conducted by municipalities in Japan only involves cytology (Pap smear) and not primary HPV testing. Given these issues, large‐scale epidemiological studies may not be capable of accurately evaluating the effectiveness of the HPV vaccine in Japan.

Since 2014, we have been conducting a large‐scale epidemiological study in Niigata Prefecture aimed at overcoming the aforementioned challenges and obtaining an accurate assessment of HPV vaccine effectiveness in Japanese women (the Niigata study). In the Niigata study, official municipal vaccination records are checked, HPV testing is performed on registrants during cervical screening, and a questionnaire is used to obtain information on sexual activity. In an interim report of women aged 20–22 years, we reported bivalent vaccine effectiveness to be >90% in preventing HPV 16/18 infection.^28^, ^29^ The present study aimed to analyze the effectiveness of the HPV vaccine in preventing cervical cytological abnormalities in women aged 20–26 years, adjusted for sexual activity.

## 2 MATERIALS AND METHODS

### 2.1 Study design and target population

This population‐based cross‐sectional study (the Niigata study) comprised women aged 20–26 years (born in fiscal years 1992–1999) who have undergone cervical screening in six cities in Niigata Prefecture (Niigata, Nagaoka, Joetsu, Shibata, Sanjo, and Mitsuke) since April 2014.^28^ Women born between April 1994 and March 2000 (fiscal years 1994–1999) were eligible to receive free HPV vaccination (50% subsidized by the central government; 50% subsidized by the municipality), starting in October 2010. Vaccination coverage in this age group is >70%.^8^, ^9^, ^10^ In 2010, the cities of Niigata, Nagaoka, Joetsu, Shibata, and Mitsuke introduced public subsidies for girls born in or after 1994. In 2012, the city of Sanjo introduced public subsidies for girls born in or after 1996. The number of cases needed to examine the effectiveness of the HPV vaccine against the cytological abnormality of a high‐grade squamous intraepithelial lesion (HSIL) or worse (HSIL+) was calculated as follows. Based on data from our previous research in Niigata Prefecture, we assumed that HSIL+ rates among unvaccinated women and women who had been vaccinated prior to sexual debut would be 0.66% and 0%, respectively, at a ratio of 1:2.2 (unvaccinated to vaccinated). With a two‐sided significance of 0.05 and a power of 0.8, we calculated that a total of 2966 participants were needed for analysis in this study.

### 2.2 Vaccination status and sexual history

Japan does not have a national vaccination database, and vaccination records are managed by individual municipalities. Therefore, we obtained information on the date of vaccination, the number of doses administered, and the type of vaccine from the official vaccination records kept by the aforementioned six cities. Some registrants may have been vaccinated in a different municipality from their place of residence. Therefore, we also used a questionnaire to obtain their HPV vaccination status, along with information concerning sexual activity (age at sexual debut and number of previous sexual partners). The number of sexual partners was separated into the following five categories: 0, 1, 2–5, 6–9, and ≥ 10. We extracted “the vaccinated prior to sexual debut group” from the overall vaccinated group according to information on the date of vaccination and age at sexual debut.

### 2.3 Cervical cancer screening and human papillomavirus testing

Cervical cytology was performed using liquid‐based samples (SurePath BD Diagnostics, Sparks, MD) at the facilities where the registrants underwent screening. Data used in the analysis comprised the screening results recorded by each municipality. HPV testing was performed using residual fluid from the sample used for cervical screening. The HPV screening test was performed using HC2 (Qiagen, Hilden, Germany) or a BD Onclarity HPV kit (BD, New Jersey, United States), and positive screened samples were tested for genotype using the MEBGEN HPV kit (MBL, Nagoya, Japan). Genotype testing can detect 13 types of high‐risk HPV: 16, 18, 31, 33, 35, 39, 45, 51, 52, 56, 58, 59, and 68.

### 2.4 Statistical analyses

Data were analyzed using EZR (Jichi Medical University Saitama Medical Center), a graphical user interface of R (R Foundation for Data Computing, Vienna, Austria).^30^ Categorical data were expressed as the overall number and percentage, and continuous data were expressed as the median or mean value. The age at sexual debut was analyzed as a continuous variable without grouping, and the number of sexual partners was analyzed in five groups: 0, 1, 2–5, 6–9, and 10 or more. Among the registrants, there was one woman who indicated that her age at sexual debut was less than 10 years old (age 8 years). To minimize the influence of outliers, this woman was excluded from the analysis. The Mann–Whitney *U*, Pearson’s χ^2^, and Fisher’s exact tests were used to compare baseline characteristics, HPV infection rates, and abnormal cytology rates in vaccinated and unvaccinated women. Univariate and multivariate logistic regression analyses were performed to evaluate vaccine effectiveness (VE) for HPV 16/18 infection and HSIL+. In the multivariate logistic regression analysis, Model 1 was adjusted for age and Model 2 was adjusted for age and total number of previous sexual partners. We set the “previous sexual partner = 0” group as the reference in Model 2. VE was calculated as 100 × [1 − odds ratio] along with the corresponding 95% confidence interval (CI). A *P‐*value <0.05 was considered statistically significant.

### 2.5 Ethical statement

All registrants provided written informed consent. HPV testing, which is not part of Japan’s cervical screening program, was provided free of charge. This study was approved by the Ethical Review Board of the Niigata University Graduate School of Medical and Dental Sciences (2015–2268) and registered with the University Hospital Medical Information Network Clinical Trials Registry (UMIN) (UMIN000026769).

## 3 RESULTS

### 3.1 Classification of human papillomavirus vaccination status

**FIGURE 1:**
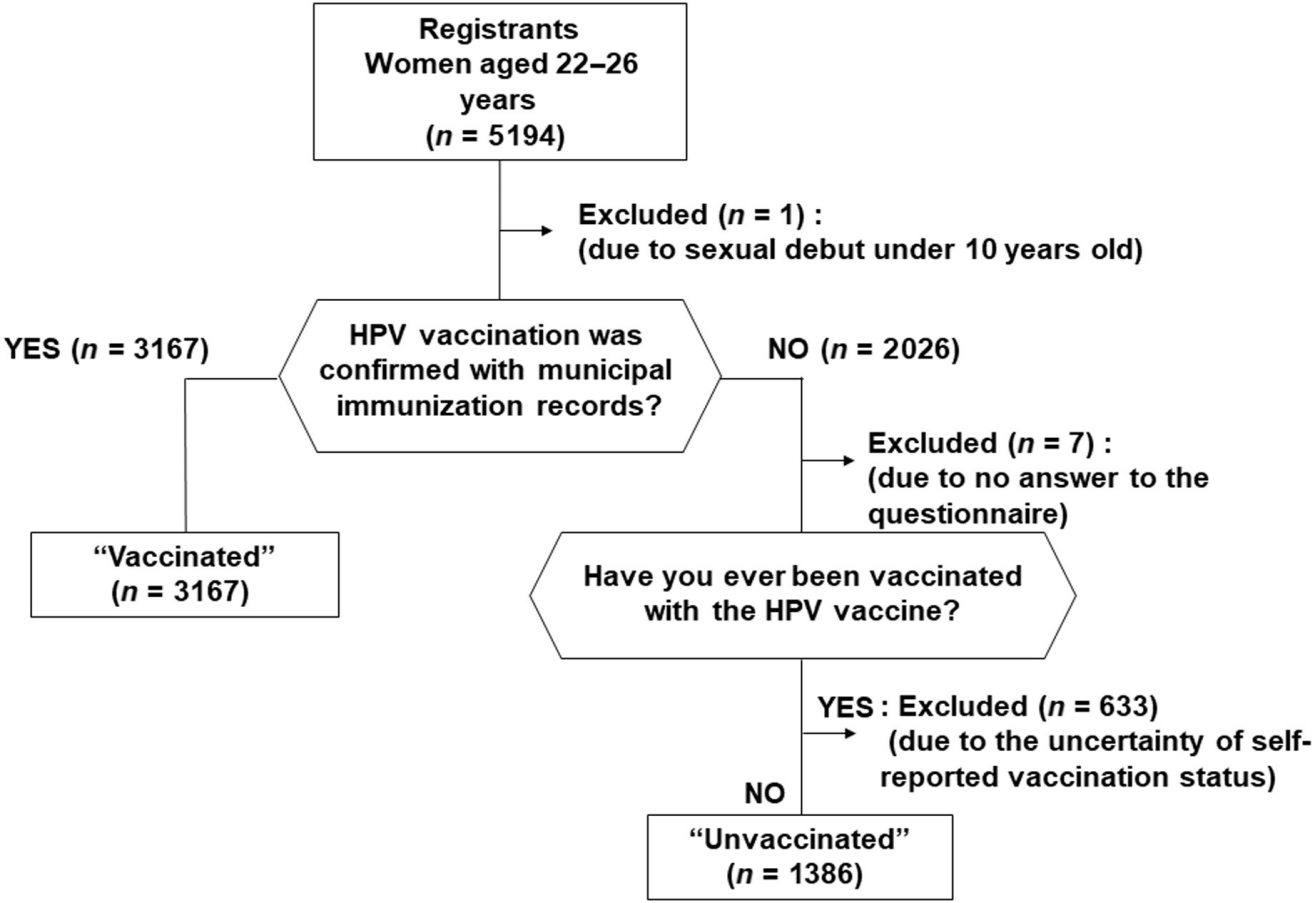
Classification of human papillomavirus (HPV) vaccination status based on municipal immunization records and self‐reporting. Of the 5194 participants, 3167 were officially confirmed to have received the HPV vaccine. Of the 2026 women who did not have a record of HPV vaccination, 1386 women self‐reported that they had not been vaccinated. Therefore, the final analysis included 3167 women in the vaccinated group and 1386 women in the unvaccinated group

Figure 1 shows the registrants’ vaccination status. Of 5194 women registered for this study, the HPV vaccination status of 3167 women could be confirmed in official municipal vaccination records. Of 2026 women with no municipal record of ever having received an HPV vaccine, 1386 women who self‐reported not having had the HPV vaccine in the questionnaire were classified as unvaccinated, while 633 women who self‐reported having been vaccinated in the questionnaire were assessed as having an uncertain vaccination status and were excluded from the analysis. Therefore, the final analysis included 3167 women in the vaccinated group and 1386 women in the unvaccinated group, comprising 4553 women in total.

### 3.2 Registrants’ baseline characteristics

Table 1 shows the registrants’ baseline characteristics. The median age (range) at registration was 21 (19–25) years in the vaccinated group and 24 (19–26) years in the unvaccinated group (*P <* 0.001). In the vaccinated group (*n* = 3167), mean age (±standard deviation [*SD*]) at first vaccination was 14.2 (±1.5) years. Of these registrants, 2821 (89.0%) women had been vaccinated prior to sexual debut, and 347 (11.0%) had their sexual debut prior to their first vaccination or at the same age. In total, 3026 (95.5%) women had completed all three vaccinations, and 2822 (89.1%) had received the bivalent vaccine. Number of sexual partners was found to be significantly higher in the unvaccinated group (*P <* 0.001). The percentage of women who responded that they had ≥10 sexual partners was 7.4% (235 of 3168) in the vaccinated group and 14.3% (198 of 1386) in the unvaccinated group. Further, the median age (range) at sexual debut was 18 (12–24) years in the vaccinated group and 18 (11–26) years in the unvaccinated group. Although the median age at sexual debut was 18 years for both groups, there were statistically significant differences in the age distribution (*P =* 0.022).

**TABLE 1:** Study registrants’ characteristics

	Vaccinated (*n* = 3167)		Unvaccinated (*n* = 1386)		*P‐*value
Age at registration					
Median (range)	21 (19–25)		24 (19–26)		<0.001^a^
Age at first inoculation					
Mean (±SD)	14.2 (±1.5)		NE		NE
Age at sexual debut					
Median (range)	18 (12–24)		18 (11–26)		0.022^a^
Timing of sexual debut (%)					
After vaccination	2821	(89.1%)	NE		NE
Same year as vaccination	205	(6.5%)	NE		
Before vaccination	141	(4.5%)	NE		
Dose of vaccine (%)					
Three	3025	(95.5%)	NE		NE
Two	102	(3.2%)	NE		
One	40	(1.3%)	NE		
Types of vaccine (%)					
Bivalent	2821	(89.1%)	NE		NE
Quadrivalent	346	(10.9%)	NE		
Number of previous sexual partners					
0	603	(19.0%)	104	(7.5%)	<0.001^b^
1	889	(28.1%)	298	(21.5%)	
2–5	1217	(38.4%)	597	(43.1%)	
6–9	223	(7.0%)	189	(13.6%)	
≥10	235	(7.4%)	198	(14.3%)	

NE, not evaluated.

^a^ Mann–Whitney *U*‐test.

^b^ χ^2^ test.

### 3.3 Prevalence of human papillomavirus infection and cytological abnormalities according to vaccination status

Table 2 shows the differences in HPV infection rates and cytological abnormalities depending on vaccination status. The vaccinated group included all women who had received at least one dose of the HPV vaccine. The high‐risk HPV positive rates in the vaccinated and unvaccinated groups were 10.4% (328 of 3167) and 17.3% (240 of 1386), respectively. The HPV 16/18 positive rates in the vaccinated and unvaccinated groups were 0.2% (*n* = 7) and 3.5% (*n* = 48), respectively. Both positive rates were significantly lower in the vaccinated group (*P <* 0.001). Atypical squamous cells of undetermined significance (ASC‐US) or worse (ASC‐US+) were observed in 148 (4.7%) women in the vaccinated group and in 101 (7.3%) women in the unvaccinated group, respectively. A high‐grade squamous intraepithelial lesion (HSIL) or worse (HSIL+) was observed in 11 (0.3%) women in the vaccinated group and in 13 (0.9%) women in the unvaccinated group. Both findings indicated a significant reduction in the vaccinated group (*P <* 0.001 and *P =* 0.013, respectively). The HPV 31/45/52 and HPV 31/33/45/52/58 positive rates were 3.4% (*n* = 107) and 5.0% (*n* = 157) in the vaccinated group, and 6.6% (*n* = 91) and 9.3% (*n* = 129) in the unvaccinated group, respectively. Both positive rates were significantly lower in the vaccinated group (*P <* 0.001). HPV 31/45/52 are cross‐protective types of the bivalent vaccine for Japanese women^28^, ^29^ and HPV 31/33/45/52/58 are vaccine types included in the 9‐valent vaccine.

**TABLE 2:** Prevalence of HPV infection and cytological abnormality according to HPV vaccination status

	Vaccinated		Unvaccinated		*P‐*value
	(*n* = 3167)		(*n* = 1386)		
HPV infection					
High‐risk HPV^a^	328	(10.4%)	240	(17.3%)	<0.001^b^
HPV 16/18	7	(0.2%)	48	(3.5%)	<0.001^b^
HPV 31/45/52	107	(3.4%)	91	(6.6%)	<0.001^b^
HPV 31/33/45/52/58	157	(5.0%)	129	(9.3%)	<0.001^b^
Cytology					
NILM	3019	(95.3%)	1285	(92.7%)	<0.001^c^
ASC‐US	50	(1.6%)	48	(3.5%)	
ASC‐H	2	(0.1%)	6	(0.4%)	
LSIL	85	(2.7%)	34	(2.5%)	
HSIL	11	(0.3%)	13	(0.9%)	
ASC‐US+	148	(4.7%)	101	(7.3%)	<0.001^b^
HSIL+	11	(0.3%)	13	(0.9%)	0.013^b^

ASC‐H, atypical squamous cells, cannot rule out high grade squamous intra‐ epithelial lesion; ASC‐US, atypical squamous cells of undetermined significance; ASC‐US+, ASC‐US or worse; HPV, human papillomavirus; HSIL, high‐grade squamous intraepithelial lesion; HSIL+, HSIL or worse; LSIL, low‐grade squamous intraepithelial lesion; NILM, negative for intraepithelial lesion or malignancy.

^a^ HPV 16/18/31/33/35/39/45/51/52/56/58/59/68.

^b^ Fisher’s exact test.

^c^ χ^2^ test.

**FIGURE 2:**
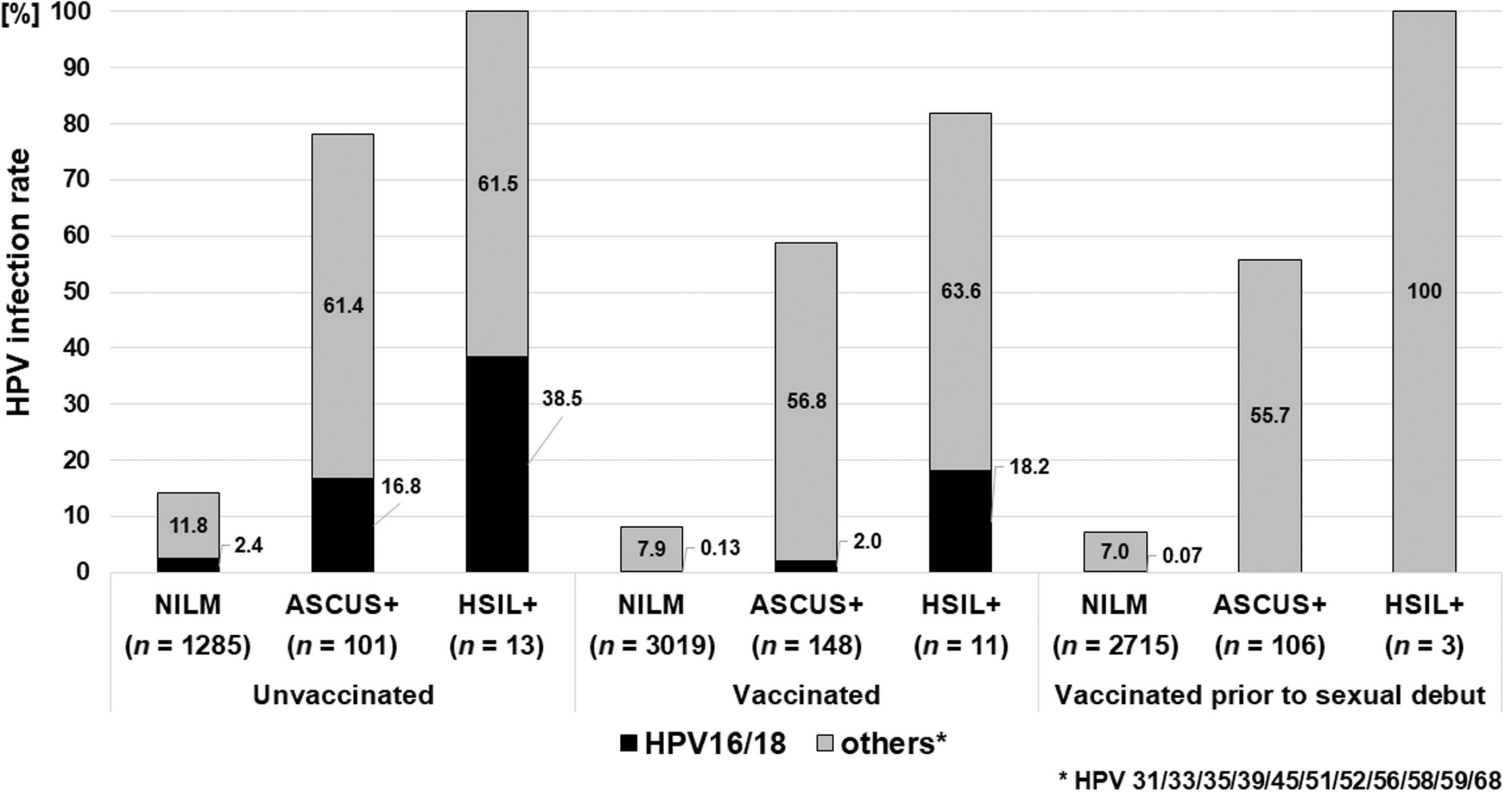
Human papillomavirus (HPV) infection rate in women with abnormal cytology by HPV vaccination status. The proportion of HPV16/18‐infected patients in the ASC‐US+ was 2.0% (3/148) in the vaccinated group compared with the 16.8% (17/101) in the unvaccinated group. The proportion of HPV 16/18‐infection in those with HSIL+ was 18.2% (2/11) in the vaccinated group compared with the 38.5% (5/13) in the unvaccinated group. None of the cytological abnormalities associated with HPV16/18 infection were observed in the vaccinated group before sexual debut

Figure 2 shows the difference in HPV infection rates depending on vaccination status in cases with cytological abnormalities. The proportion of HPV 16/18‐infected registrants who were ASC‐US+ was 2.0% (3 of 148) in the vaccinated group and 16.8% (17 of 101) in the unvaccinated group. The proportion of HPV 16/18 infected registrants who were HSIL+ was 18.2% (2 of 11) in the vaccinated group and 38.5% (5 of 13) in the unvaccinated group. When analysis was restricted to women who had been vaccinated prior to sexual debut, none of the vaccinated group exhibited cytological abnormalities related to HPV 16/18 infection. Table S1 shows the detailed prevalence of HPV type‐specific infections according to the presence or absence of cytological abnormalities, stratified by HPV vaccination status.

### 3.4 Vaccine effectiveness against human papillomavirus infection and cytological abnormality

Table 3 shows HPV vaccine effectiveness against HPV 16/18 infection and HSIL+ in all vaccinated women. In terms of effectiveness in preventing HPV 16/18 infection, VE was 94.1% (95% CI 87.0–97.3%; *P <* 0.001) in the univariate analysis, 91.4% (95% CI 79.5–96.4%; *P <* 0.001) in Model 1 (adjusted for age), and 88.8% (95% CI 73.4–95.3%; *P <* 0.001) in Model 2 (adjusted for age and number of sexual partners), showing significant effectiveness in all analyses. In terms of effectiveness against HSIL+, VE was 64.0% (95% CI 19.4–83.9%; *P =* 0.013) in the univariate analysis, 54.1% (95% CI −21.0–82.6%; *P =* 0.116) in Model 1, and 41.1% (95% CI ‐53.0–77.3%; *P =* 0.276) in Model 2, with the multivariate analyses adjusted for age and number of sexual partners not showing significance.

**TABLE 3:** Vaccine effectiveness against HPV infection and cytological abnormality

	HPV 16/18 infection			HSIL+		
Crude analysis^a^						
OR (95% CI)	0.06	(0.03–0.13)	*P <* 0.001	0.36	(0.16–0.81)	*P =* 0.013
VE (95% CI)	94.1	(87.0–97.3)		64.0	(19.4–83.9)	
Model 1^a^						
aOR (95% CI)	0.09	(0.04–0.21)	*P <* 0.001	0.46	(0.17–1.21)	*P =* 0.116
aVE (95% CI)	91.4	(79.5–96.4)		54.1	(−21.0–82.6)	
Model 2^a^						
aOR (95% CI)	0.11	(0.05–0.27)	*P <* 0.001	0.59	(0.23–1.53)	*P =* 0.276
aVE (95% CI)	88.8	(73.4–95.3)		41.1	(−53.0–77.3)	

*Note*: Model 1: adjusted for age.

*Note*: Model 2: adjusted for age and number of lifetime sexual partners.

aOR, adjusted odds ratio; aVE, adjusted vaccine effectiveness; CI, confidence interval; HPV, human papillomavirus; HSIL, high‐grade squamous intraepithelial lesion; HSIL+: HSIL or worse; OR, odds ratio; VE, vaccine effectiveness.

^a^ Vaccinated *vs* unvaccinated (logistic regression test).

### 3.5 Vaccine effectiveness against human papillomavirus infection and cytological abnormality in vaccinated women prior to sexual debut

It is recommended that the HPV vaccine be administered prior to sexual debut to maximize its effectiveness. Therefore, we have added an additional analysis of the vaccine effectiveness against cytological abnormalities in vaccinated women prior to sexual debut, in addition to a comparison with all vaccinated women. Table 4 shows the effectiveness of the vaccine against HPV 16/18 infection and HSIL+ only among women who had been vaccinated prior to sexual debut. VE for HPV 16/18 infection was 98.1% (95% CI 92.3–99.5%; *P <* 0.001) in the univariate analysis, 97.4% (95% CI 88.6–99.4%; *P <* 0.001) in Model 1 and 95.8% (95% CI 81.7–99.0%; *P <* 0.001) in Model 2. VE for HSIL+ was 89.1% (95% CI 61.6–96.9%; *P <* 0.001) in the univariate analysis, 86.2% (95% CI 43.9–96.6%; *P =* 0.006) in Model 1, and 78.3% (95% CI 11.3–94.7%; *P =* 0.033) in Model 2, showing significant effectiveness in all analyses, especially multivariate analyses adjusted for age and the number of sexual partners.

**TABLE 4:** Vaccine effectiveness against HPV infection and cytological abnormality in vaccinated women prior to sexual debut

	HPV 16/18 infection			HSIL+		
Positive rate						
Vaccinated^a^ (*n* = 2821)	2 (0.1%)			3 (0.1%)		
Unvaccinated (*n* = 1386)	48 (3.5%)			13 (0.9%)		
Crude analysis^b^						
OR (95% CI)	0.02	(0.00–0.08)	*P <* 0.001	0.11	(0.03–0.38)	*P <* 0.001
VE (95% CI)	98.1	(92.3–99.5)		89.1	(61.6–96.9)	
Model 1^b^						
aOR (95% CI)	0.03	(0.01–0.11)	*P <* 0.001	0.14	(0.03–0.56)	*P =* 0.006
aVE (95% CI)	97.4	(88.6–99.4)		86.2	(43.9–96.6)	
Model 2^b^						
aOR (95% CI)	0.04	(0.01–0.18)	*P <* 0.001	0.22	(0.05–0.89)	*P =* 0.033
aVE (95% CI)	95.8	(81.7–99.0)		78.3	(11.3–94.7)	

*Note*: Model 1: adjusted for age.

*Note*: Model 2: adjusted for age and number of lifetime sexual partners.

aOR, adjusted odds ratio; aVE, adjusted vaccine effectiveness; CI, confidence interval; HPV, human papillomavirus; HSIL, high‐grade squamous intraepithelial lesion; HSIL+, HSIL or worse; OR, odds ratio; VE, vaccine effectiveness.

^a^ Vaccinated women prior to sexual debut.

^b^ Vaccinated *vs* unvaccinated (logistic regression test).

Because vaccinated women prior to sexual debut include women who have not had sexual intercourse, we excluded the “previous sexual partner = 0” group from the “after vaccination” group for our statistical analysis. The corrected results, which were almost identical to the previous results, are added as Tables S2 and S3.

## 4 DISCUSSION

This study is the first in Japan to evaluate the effectiveness of HPV vaccination against cervical cytological abnormalities using accurate municipal vaccination data and taking HPV infection status and sexual activity into account. Using multivariate analyses adjusted for age and the number of sexual partners, we found a significant preventive effect against high‐grade cytological abnormalities (HSIL+) for women who had received the HPV vaccine prior to sexual debut.

This study had the following three strengths. First, the analysis used accurate vaccination information obtained from municipalities (date of vaccination, type of vaccine, and number of doses administered). Second, the questionnaire survey of sexual activity allowed us to adjust for the number of sexual partners in the multivariate analysis and restrict some analyses to women who had been vaccinated prior to sexual debut. Adjusting for sexual activity, which is a confounding factor that has a considerable effect on HPV infection, has not been performed in most previous analyses from Japan and is likely to enhance the accuracy of the present study’s findings on the effectiveness of the HPV vaccine. Third, HPV testing was performed at the same time as cytology examinations. Understanding HPV infection status made it possible to analyze cytological abnormalities associated with the HPV types targeted using the vaccines.

In an interim analysis as part of the Niigata study, we examined the effectiveness of the bivalent vaccine for preventing HPV 16/18 infection in 1814 women aged 20–22 years in Niigata Prefecture between fiscal years 2014 and 2016. We found that VE was 91.9% in the HPV vaccinated group overall and 93.9% in the group vaccinated prior to sexual debut.^28^ In this present study, we registered cases for an additional 3 years and observed a similar positive effect on preventing HPV 16/18 infection in 4554 women aged 20–26 years. A previous study that investigated the reliability of self‐reported vaccination data found that, with respect to municipal vaccination records, self‐reports had 85.2% sensitivity, 59.8% specificity, 87.7% positive predictive value, and 54.5% negative predictive value.^27^ Self‐reports did not match municipal records in 20.6% of cases. Moreover, approximately 50% of women who had self‐reported not having been vaccinated had actually been vaccinated but had forgotten.^27^ Japan comprises many local governments, all of whom manage their own vaccination records and cervical screening results. Therefore, due to the lack of a national registration system for vaccination and cervical screenings, and because personal information is not centrally managed, conducting large‐scale epidemiological research at a national level is challenging. Therefore, previous studies concerning the effectiveness of the HPV vaccines in Japan have used self‐reported vaccination data. Moreover, because HPV testing does not form part of general cervical cancer screening in Japan, the HPV infection status is not included in the analyses of cancer screening data.

Using Miyagi Prefecture cervical cancer screening data, Karube et al. reported that the rate of HSIL+ was 0.20% (2 of 1002) in vaccinated women and 1.14% (56 of 4922) in unvaccinated women, indicating a significant reduction of 82.5% due to vaccination (*P <* 0.0001).^22^ Using screening data from Akita Prefecture, Tanaka et al. reported that the rate of ASC‐US+ was significantly lower in vaccinated women at 0.24% (1 of 413) compared with 2.0% (41 of 2012) in unvaccinated women (*P =* 0.01); however, the difference in HSIL+ was not significant (*P =* 0.596).^23^ These studies on cytological abnormality used self‐reported vaccination data and did not examine municipal records. Regarding reductions in histological abnormalities, Shiko et al. reported that VE against CIN2+ was 76% (relative risk 0.24, 95% CI 0.10–0.60).^24^ Our results showed that the proportion of HPV 16/18 among cases with HSIL+ decreased from 38.5% in the unvaccinated group to 0% in the group vaccinated prior to sexual debut. Matsumoto et al. conducted a prospective study and registered cases with cervical histological abnormalities for 6 years, starting in 2012. In that study, the proportion of CIN2+/AIS due to HPV 16/18 decreased from 43.5% (10 of 23) in unvaccinated women to 12.5% (2 of 16) in vaccinated women.^31^ The aforementioned study of histological abnormalities also did not use vaccination data from municipal records.

Using municipal vaccination records, Ueda et al. conducted a survey concerning the low‐grade squamous intraepithelial lesion cytological abnormality (LSIL+) rate in 20‐year‐old women who had undergone cervical cancer screening. The LSIL+ rate was significantly lower among women young, enough to be eligible for vaccination at 0.58% (6 of 1032) compared with women from the generations before vaccination was introduced (2.11% [60 of 2841], *P <* 0.01).^25^ Ikeda et. al reported that the odds ratio for vaccine effectiveness against CIN2+ was 0.25 (95% CI 0.12–0.54) in women who had undergone cervical cancer screening in 31 municipalities.^26^ These studies obtained accurate vaccination data through checking municipal vaccination records; however, data concerning HPV infection status or sexual activity were not included in their analyses. As described above, previous analyses of vaccine effectiveness in Japan have not considered differences in sexual activity, leaving any associations with being vaccinated prior to or after sexual debut unclear.

The World Health Organization (WHO) has recommended that girls become vaccinated for HPV before they are sexually active,^32^ and our findings support this recommendation for Japanese women. The WHO recommends vaccinating girls aged 9–14 years; however, the age range in Japan for routine vaccination is 12–16 years. In our previous research, although the mean age of sexual debut for Japanese women is 18.4 years, 24.1% underwent sexual debut at ages 15–16 years and 3.4% at age ≤ 14.^33^ These findings indicate that the recommended age for vaccination in Japan should be ≤14 years. Recently the Japanese Government decided to resume proactive recommendations for the HPV vaccine.^34^ Consequently, vaccination coverage needs to be rapidly increased via catch‐up vaccinations, and discussion concerning the optimal age for publicly funded vaccination programs is needed.

This study had some limitations. First, the data are only derived from a single region of Japan (Niigata Prefecture). Niigata is located on the northwest coast of Honshu, Japan’s largest island, and its current population is approximately 2,200,000. The six cities that participated in the study account for approximately 70% of the population of Niigata and are representative of the prefecture’s municipalities. The mean annual income in Niigata is 5,331,000 yen (US$48,300), which is similar to the mean annual income of Japan overall (5,382,000 yen, US$48,900). Thus, it can be considered a socially average prefecture in Japan.^35^ However, limiting the study to the single region of Niigata Prefecture had some advantages: the registrants shared a similar social context, personal information could be obtained directly from municipal authorities, and a detailed questionnaire survey could be conducted. The second issue is sample size. At the time of the conception of the study design, the required number of cases was set by limiting the target vaccinated group prior to sexual debut, resulting in sufficient power of 0.881 for analysis of statistically significant vaccine effectiveness. However, statistical power of 0.523 for analysis of the entire vaccinated group (assuming HSIL+0.3% in the vaccinated group, HSIL+0.9% in the unvaccinated group, and a two‐tailed significant difference of 0.05) is needed. Approximately 2000 more registrants are needed to perform an analysis with adequate power to avoid type one and type two errors for the entire vaccinated group.

Recently, we reported that the HPV 16/18 infection rates were increasing again due to discontinuation of active recommendations for the HPV vaccine^36^ and that the HPV vaccine demonstrated a long‐term preventive effect against HPV 16/18 infection even 9 years post‐vaccination.^37^, ^38^ After proactive recommendation for HPV vaccine resumes, HPV vaccination coverage is expected to increase in Japan. We plan to continue registering cases for the Niigata study in the future.

We found that among Japanese women aged 20–26 years, a high VE of >90% was observed concerning HPV 16/18 infection and that VE was nearly 80% for HSIL+ cytological abnormalities in the group vaccinated prior to sexual debut using real‐world data. Japanese girls aged 12–16 years are offered free HPV vaccination; however, the WHO recommends vaccinating girls aged 9–14 years. After proactive recommendation for HPV vaccine resumes, it will be necessary to discuss the recommended age for routine HPV vaccination in Japan based on our results. As part of the Niigata study, we plan to further verify the effectiveness of the vaccines against histological abnormalities and analyze their long‐term prevention effectiveness.

## CONFLICT OF INTEREST

M. Y., Y. U., and T. E. have received lecture fees from Merck Sharp and Dohme. E. M. received honoraria and lecture fees from Roche Diagnostics, Hologic Japan, and Merck Sharp and Dohme. All other authors report no potential conflicts.

## ETHICS STATEMENT

Approval of the research protocol by an Institutional Reviewer Board: This study was approved by the Ethical Review Board of the Niigata University Graduate School of Medical and Dental Sciences (2015–2268). Informed Consent: All registrants provided written, informed consent. Registry and the Registration No. of the study/trial: This study was registered with the University Hospital Medical Information Network Clinical Trials Registry (UMIN) (UMIN000026769). **Animal Studies:** N/A.

## Supporting information


Table S1
Table S2
Table S3
