# Supplementary material for: Effectiveness of human papillomavirus vaccine against cervical precancer in Japan: Multivariate analyses adjusted for sexual activity
Source: Cancer Sci. 2022 Jul 11;113(9):3211–20. doi: 10.1111/cas.15471 (PMC9459348; doi:10.1111/cas.15471)
Supplement: Supplementary file 1 — Table S1 Table S2 Table S3 [file CAS-113-3211-s001.docx]

**Supplementary Table1**

Prevalence of HPV type specific infection and cytological abnormality according to HPV vaccination status

|  | **Unvaccinated (n = 1386)** | | | | | |  | **Vaccinated (n = 3167)** | | | | | |  | **Vaccinated prior to sexual debut (n = 2821)** | | | | | |
| --- | --- | --- | --- | --- | --- | --- | --- | --- | --- | --- | --- | --- | --- | --- | --- | --- | --- | --- | --- | --- |
|  | **NILM (n=1285)** | | **ASC-US+ (n=101)** | | **HSIL+**  **(n=13)** | |  | **NILM (n=3019)** | | **ASC-US+ (n=148)** | | **HSIL+**  **(n=11)** | |  | **NILM (n=2715)** | | **ASC-US+ (n=106)** | | **HSIL+**  **(n=3)** | |
| **HPV16** | 24 | (1.9%) | 14 | (13.9%) | 4 | (30.8%) |  | 3 | (0.1%) | 3 | (2.0%) | 2 | (18.2%) |  | 2 | (0.1%) | 0 | (0.0%) | 0 | (0.0%) |
| **HPV18** | 7 | (0.5%) | 4 | (4.0%) | 1 | (7.7%) |  | 1 | (0.0%) | 0 | (0.0%) | 0 | (0.0%) |  | 0 | (0.0%) | 0 | (0.0%) | 0 | (0.0%) |
| **HPV31** | 18 | (1.4%) | 7 | (6.9%) | 2 | (15.4%) |  | 5 | (0.2%) | 2 | (1.4%) | 2 | (18.2%) |  | 1 | (0.0%) | 0 | (0.0%) | 0 | (0.0%) |
| **HPV33** | 2 | (0.2%) | 5 | (5.0%) | 0 | (0.0%) |  | 6 | (0.2%) | 0 | (0.0%) | 0 | (0.0%) |  | 6 | (0.2%) | 0 | (0.0%) | 0 | (0.0%) |
| **HPV35** | 2 | (0.2%) | 3 | (3.0%) | 0 | (0.0%) |  | 5 | (0.2%) | 3 | (2.0%) | 1 | (9.1%) |  | 3 | (0.1%) | 1 | (0.9%) | 0 | (0.0%) |
| **HPV39** | 20 | (1.6%) | 4 | (4.0%) | 0 | (0.0%) |  | 29 | (1.0%) | 5 | (3.4%) | 0 | (0.0%) |  | 21 | (0.8%) | 2 | (1.9%) | 0 | (0.0%) |
| **HPV45** | 4 | (0.3%) | 2 | (2.0%) | 0 | (0.0%) |  | 0 | (0.0%) | 0 | (0.0%) | 0 | (0.0%) |  | 0 | (0.0%) | 0 | (0.0%) | 0 | (0.0%) |
| **HPV51** | 29 | (2.3%) | 10 | (9.9%) | 1 | (7.7%) |  | 43 | (1.4%) | 15 | (10.1%) | 1 | (9.1%) |  | 36 | (1.3%) | 12 | (11.3%) | 1 | (33.3%) |
| **HPV52** | 55 | (4.3%) | 11 | (10.9%) | 3 | (23.1%) |  | 76 | (2.5%) | 26 | (17.6%) | 3 | (27.3%) |  | 63 | (2.3%) | 20 | (18.9%) | 0 | (0.0%) |
| **HPV56** | 26 | (2.0%) | 15 | (14.9%) | 0 | (0.0%) |  | 42 | (1.4%) | 31 | (20.9%) | 2 | (18.2%) |  | 33 | (1.2%) | 22 | (20.8%) | 1 | (33.3%) |
| **HPV58** | 30 | (2.3%) | 19 | (18.8%) | 4 | (30.8%) |  | 41 | (1.4%) | 22 | (14.9%) | 3 | (27.3%) |  | 32 | (1.2%) | 15 | (14.2%) | 1 | (33.3%) |
| **HPV59** | 13 | (1.0%) | 8 | (7.9%) | 1 | (7.7%) |  | 33 | (1.1%) | 5 | (3.4%) | 0 | (0.0%) |  | 30 | (1.1%) | 3 | (2.8%) | 0 | (0.0%) |
| **HPV68** | 16 | (1.2%) | 5 | (5.0%) | 0 | (0.0%) |  | 22 | (0.7%) | 7 | (4.7%) | 1 | (9.1%) |  | 20 | (0.7%) | 3 | (2.8%) | 1 | (33.3%) |

ASC-US, atypical squamous cells of undetermined significance; ASC-US+, ASC-US or worse; HPV, human papillomavirus; HSIL, high-grade squamous intraepithelial lesion; HSIL+, HSIL or worse; NILM, negative for intraepithelial lesion or malignancy

**Supplementary Table 2**

Vaccine effectiveness against HPV infection and cytological abnormality in sexually active women

|  |  | HPV 16/18 infection | | | HSIL+ | | |  |
| --- | --- | --- | --- | --- | --- | --- | --- | --- |
|  | **Positive rate** |  |  |  |  |  |  |  |
|  | Vaccinated (n = 2565) | 7 (0.3%) | | | 11 (0.4%) | | |  |
|  | Unvaccinated (n = 1282) | 48 (3.7%) | | | 13 (1.0%) | | |  |
|  |  |  |  |  |  |  |  |  |
|  | **Crude analysis*** |  |  |  |  |  |  |  |
|  | OR (95% CI) | 0.07 | (0.03-0.15) | *p* < 0.001 | 0.41 | (0.19-0.93) | *p* = 0.032 |  |
|  | VE (95% CI) | 93.2 | (84.9-96.9) |  | 58.7 | (7.5-81.5) |  |  |
|  | **Model 1^†^** |  |  |  |  |  |  |  |
|  | aOR (95% CI) | 0.09 | (0.04-0.22) | *p* < 0.001 | 0.48 | (0.18-1.26) | *p* = 0.135 |  |
|  | aVE (95% CI) | 90.8 | (78.2-96.1) |  | 51.9 | (-26.0-81.6) |  |  |
|  | **Model 2^†^** |  |  |  |  |  |  |  |
|  | aOR (95% CI) | 0.11 | (0.05-0.27) | *p* < 0.001 | 0.59 | (0.23-1.53) | *p* = 0.276 |  |
|  | aVE (95% CI) | 88.8 | (73.4-95.3) |  | 41.1 | (-53.0-77.3) |  |  |
|  |  |  |  |  |  |  |  |  |

*vaccinated vs. unvaccinated (logistic regression test)

Model 1: adjusted for age

Model 2: adjusted for age and number of lifetime sexual partners

aOR, adjusted odds ratio; aVE: adjusted vaccine effectiveness; CI, confidence interval; HPV, human papillomavirus; HSIL, high-grade squamous intraepithelial lesion; HSIL+: HSIL or worse; OR, odds ratio; VE, vaccine effectiveness

**Supplementary Table 3**

Vaccine effectiveness against HPV infection and cytological abnormality in vaccinated women prior to sexual debut in sexually active women

|  |  | HPV 16/18 infection | | | HSIL+ | | |  |
| --- | --- | --- | --- | --- | --- | --- | --- | --- |
|  | **Positive rate** |  |  |  |  |  |  |  |
|  | Vaccinated* (n = 2218) | 2 (0.1%) | | | 3 (0.1%) | | |  |
|  | Unvaccinated (n = 1282) | 48 (3.7%) | | | 13 (1.0%) | | |  |
|  |  |  |  |  |  |  |  |  |
|  | **Crude analysis^†^** |  |  |  |  |  |  |  |
|  | OR (95% CI) | 0.02 | (0.00-0.09) | *p* < 0.001 | 0.13 | (0.04-0.45) | *p =* 0.001 |  |
|  | VE (95% CI) | 97.8 | (90.9-99.5) |  | 87.1 | (54.6-96.3) |  |  |
|  | **Model 1^†^** |  |  |  |  |  |  |  |
|  | aOR (95% CI) | 0.03 | (0.01-0.13) | *p* < 0.001 | 0.15 | (0.04-0.61) | *p* = 0.008 |  |
|  | aVE (95% CI) | 97.1 | (87.5-99.3) |  | 84.9 | (39.0-96.2) |  |  |
|  | **Model 2^†^** |  |  |  |  |  |  |  |
|  | aOR (95% CI) | 0.04 | (0.01-0.18) | *p* < 0.001 | 0.22 | (0.05-0.89) | *p* = 0.033 |  |
|  | aVE (95% CI) | 95.8 | (81.7-99.0) |  | 78.3 | (11.3-94.7) |  |  |
|  |  |  |  |  |  |  |  |  |

*vaccinated women prior to sexual debut

^†^vaccinated vs. unvaccinated (logistic regression test)

Model 1: adjusted for age

Model 2: adjusted for age and number of lifetime sexual partners

aOR, adjusted odds ratio; aVE, adjusted vaccine effectiveness; CI, confidence interval; HPV, human papillomavirus; HSIL, high-grade squamous intraepithelial lesion; HSIL+, HSIL or worse; OR, odds ratio; VE, vaccine effectiveness
